# 1987. "Association Between Integrase Inhibitor Plasma Concentrations and Body Weight Gain in Women Living with HIV"

**DOI:** 10.1093/ofid/ofad500.114

**Published:** 2023-11-27

**Authors:** Cecile D Lahiri, Julie B Dumond, Cyra Christina Mehta, Qian Yang, Anandi N Sheth, Igho Ofotokun, Craig Sykes, Mattie Hartauer, Adrian Khoei, Maria L L Alcaide, Jordan Lake, Leah H Rubin, Deborah Konkle-Parker, Adaora A Adimora, Elizabeth F Topper, Audrey L French, Jennifer Cocohoba, Seble Kassaye, Michael Augenbraun, Anjali Sharma, Phyllis C Tien

**Affiliations:** Emory University, Atlanta, GA; University of North Carolina, Eshelman School of Pharmacy, Chapel Hill, North Carolina; Emory University School of Medicine, Atlanta, Georgia; Emory University School of Medicine, Atlanta, Georgia; Emory University School of Medicine, Atlanta, Georgia; Emory University School of Medicine , Lilburn, GA; University of North Carolina, Eshelman School of Pharmacy, Chapel Hill, North Carolina; University of North Carolina, Eshelman School of Pharmacy, Chapel Hill, North Carolina; University of North Carolina, Eshelman School of Pharmacy, Chapel Hill, North Carolina; Division of Infectious Diseases, Department of Medicine, University of Miami Miller School of Medicine, Miami, Florida; University of Texas Health Science Center at Houston, Houston, TX; Johns Hopkins University School of Medicine, Baltimore, Maryland; University of Mississippi Medical Center, Jackson, MS; Department of Medicine, University of North Carolina at Chapel Hill, Chapel Hill, North Carolina; Johns Hopkins Bloomberg School of Public Health, Baltimore, Maryland; Stroger Hospital of Cook County, Chicago, Illinois; University of California San Francisco, School of Pharmacy, San Francisco, California; Georgetown University Medical Center, Washington, DC, Washington, DC; SUNY Downstate Medical University, Brooklyn, NY; Albert Einstein College of Medicine, New York, New York; Division of Infectious Diseases, Department of Medicine, University of California, San Francisco, San Francisco, California and Medical Service, Department of Veterans Affairs, San Francisco, California, San Francisco, CA

## Abstract

**Background:**

Integrase strand-transfer inhibitors (INSTIs) are associated with body weight gain among women living with HIV (WLH) in the Women’s Interagency HIV Study (WIHS). The role of antiretroviral therapy (ART) pharmacokinetics in INSTI-associated weight gain is unknown. We report the relationship between INSTI plasma concentrations and weight change in WLH.

Model-adjusted estimates of percent body weight change by integrase inhibitor plasma concentration

**Figure:**
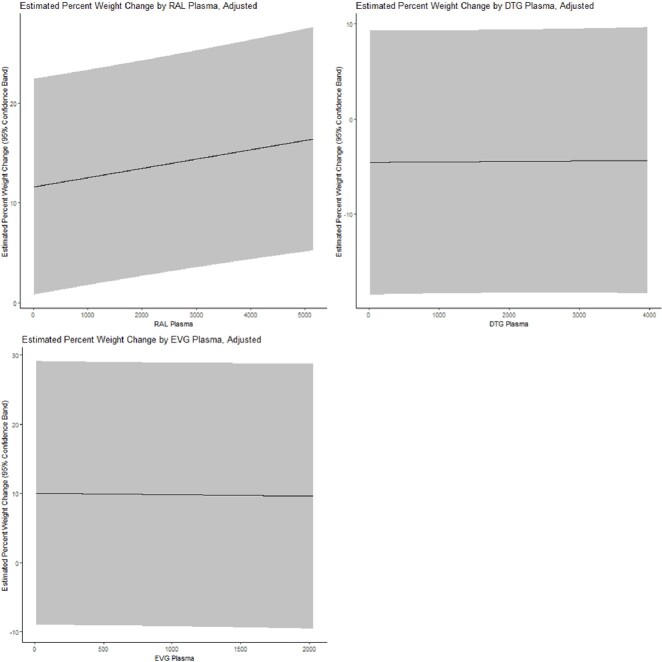

Model-adjusted estimates of percent body weight change with 95% confidence band (y-axis) by integrase inhibitor plasma concentration in ng/mL (x-axis). Models were adjusted for baseline age, race/ethnicity, baseline obesity (body mass index ≥ 30 kg/m2), history of prior non-nucleoside reverse transcriptase use, and self-reported antiretroviral therapy adherence (100% vs <100%). Abbreviations: RAL=raltegravir, DTG=dolutegravir, EVG=elvitegravir

**Methods:**

Data from 2006-2017 were analyzed from virologically suppressed (< 200 copies/mL) WLH in the WIHS who switched/added raltegravir (RAL), dolutegravir (DTG), or elvitegravir (EVG) to ART. Percent body weight change was calculated using weights obtained 6-12 months pre- and post- INSTI switch/add. INSTI random plasma concentrations were measured with validated LC/MS-MS assays 6-12 months post INSTI switch/add. Area under the curve (AUC) was calculated using estimates from published models and updating individual-level parameters with plasma concentrations. Linear models assessed the relationship between plasma concentrations or AUC and weight change for each INSTI separately. Models were adjusted for age, race/ethnicity, baseline obesity, history of non-nucleoside reverse transcriptase use, and self-reported ART adherence.

**Results:**

176 WLH contributed plasma drug concentrations with a mean ±SD 1.5 (±0.1) years follow-up: 43 (24%) were on RAL, 87 (49%) on DTG, and 47 (27%) on EVG. Mean age was 51 years (± 9), 64% were Non-Hispanic Black, baseline BMI was 31.6 kg/m^2^ (±8.8), and 57% reported 100% ART adherence. Mean percent body weight change was +2.3% (±6.0) for RAL, +1.8% (±9.0) for DTG, and +2.8% (±10.5) for EVG. AUC was able to be calculated in 167 (95%) of women. In adjusted linear models, higher RAL plasma concentration was associated with greater percent body weight change (p=0.016), **Figure 1**. No association of drug AUC with body weight change for any INSTI was observed.

**Conclusion:**

In WLH, random RAL plasma concentrations, but not the RAL AUC, was associated with body weight gain. In contrast, the effect of DTG and EVG plasma drug exposure on short-term body weight change appears to be limited. Mechanisms contributing to body weight gain may be INSTI-specific. In addition to further pharmacologic assessments, other mechanisms to explain INSTI-associated weight gain should be examined.

**Disclosures:**

**Cecile D. Lahiri, MD, MS**, Merck: Grant/Research Support|Theratechnologies, LLC: Advisor/Consultant|Theratechnologies, LLC: Honoraria **Julie B. Dumond, PharmD**, Merck: Grant/Research Support **Cyra Christina Mehta, PhD, MSPH**, Merck: Grant/Research Support **Igho Ofotokun, MD, MSc, FIDSA**, Merck: Grant/Research Support **Maria L L. Alcaide, MD**, Discidium Biosciences: Board Member|Gilead: Honoraria|Merk & Co: Honoraria|Senhwa Biosciences: Honoraria|Virology Education: Honoraria **Jordan Lake, MD**, Gilead Sciences: Grant/Research Support|Theratechnologies: Advisor/Consultant **Phyllis C. Tien, MD, MSc**, Merck: Grant/Research Support

